# Investigating the impact of COVID-19 on patients with cancer from areas of conflict within the MENA region treated at King Hussein Cancer Center

**DOI:** 10.3389/fonc.2023.1088000

**Published:** 2023-02-23

**Authors:** Maysa Al-Hussaini, Abdallah Al-Ani, Muhammad Hammouri, Leen Al-Huneidy, Asem Mansour

**Affiliations:** ^1^ Department of Pathology and Laboratory Medicine, King Hussein Cancer Center, Amman, Jordan; ^2^ Office of Scientific Affairs and Research, King Hussein Cancer Center, Amman, Jordan; ^3^ School of Medicine, The University of Jordan, Amman, Jordan; ^4^ Department of Radiology, King Hussein Cancer Center, Amman, Jordan

**Keywords:** Areas of conflict, cancer care, COVID-19, Middle East, time series analysis

## Abstract

**Background:**

There is a paucity of evidence regarding the impact of COVID-19 on cancer care among refugees or patients from areas of conflict. Cancer care for these populations remains fragmented due to resource scarcity and limited infrastructure.

**Aims:**

To explore the effect of COVID-19 on cancer care among patients from areas of conflict treated at King Hussein Cancer Center (KHCC).

**Methodology:**

We performed a retrospective chart review of all patients from areas of conflict, treated at KHCC from 2018 to 2021. Patients’ demographics and clinical characteristics are presented in the form of descriptive statistics. Interrupted Time Series (ITS) analysis was utilized to investigate the impact of COVID-19 on the number of admissions throughout the study’s period.

**Results:**

A total of 3317 patients from areas of conflict were included in the study. Among these, 1546 were males (46.6%) while 1771 (53.4%) were female. Libyans (34.6%), Palestinians (24.8%), Iraqis (24.5%), Syrians (15.3%), and Sudanese patients (0.9%) constituted our study sample. ITS analysis demonstrated that the start of the COVID-19 lockdown significantly decreased admissions by 44.0% (p = 0.020), while the end of the COVID-19 restriction significantly improved admissions by 43.0% (p = 0.023). Among those with available SEER stages, more than a quarter of patients had distant metastasis (n = 935, 28.2%) irrespective of age and biological sex. Advanced presentations during 2020 had approximately a 16% and 6% increase compared to 2018 and 2019, respectively. Breast cancer (21.4%), hematolymphoid cancers (18.1%), and cancers of the digestive system (16.5%) were the most common cancers among our cohort.

**Conclusion:**

Restrictions associated with COVID-19 had a significant effect on the number of admissions of patients from areas of conflict. In the long term, this effect may impact the survival outcomes of affected patients.

## 1 Introduction

The United Nations High Commissioner for Refugees (UNHCR) estimates that around 89.3 million people are forcibly displaced worldwide including 27.1 million refugees (1). In the Middle East, millions of Palestinian, Iraqi, Yemini, Libyan, Sudanese, and Syrian populations were displaced due to the invasions, prosecutions, and armed conflicts in the region (2). These conflicts across the region uprooted both the healthy and the ill alike. Populations from areas of conflict face significant and complicated healthcare challenges. In addition to war-related deaths and injuries, such vulnerable populations may already have diseases or develop new ones, necessitating interventions from healthcare systems, including those of their host countries. Previously, displacement crises mainly occurred in low-income and less developed countries leading to the spread of infectious diseases and malnutrition among refugees. However, in the 21^st^ century, this scenario has become more diverse and considerably long-lasting and occurs in camp and non-camp settings, as well as low and middle-income settings alike. Similar to that trend, reported data from refugee camps in Jordan showed that the main reasons for mortality among refugees were not due to infectious causes, but rather non-communicable diseases (NCDs) (3, 4). This category of diseases has been given very little attention despite their increasing burden (5).

Of all NCD diseases, cancer is among the most important in this category as it is common, leads to substantial mortality and morbidity, and has significant economic burdens on patients and healthcare systems (6). Cancer and other NCDs are critically overlooked aspects of health in areas of conflict. Although actions in those regards have the potential to prevent thousands of deaths in these populations, reasons for such neglect stem from misconceptions of cancer as being too complex and expensive to treat, lack of agreement on cost-effective measures, and the inability of host healthcare systems to adopt new cancer services with the constant underfunded humanitarian responses (7). People from areas of conflict are susceptible to developing cancer due to many hardships including low-income. In addition, they are more likely to present with advanced stages and suffer more complications because of poor hygiene and living conditions, as well as limited access to care and resources available (8). For refugees, host countries encounter serious challenges in health provision to cancer patients considering the economic pressures they face and the lack of infrastructure, all of which lead to suboptimal care delivery to this subset of patients (9, 10). Furthermore, the course of treating many types of cancer requires a robust healthcare system with the ability to provide effective screening programs, diagnostic services, and treatment modalities (e.g., surgery, radiotherapy, or chemotherapy) (11).

Furthermore, the impact of COVID-19 on the accessibility and quality of cancer care was heavily noted within literature (12). COVID-19 has affected the entire spectrum of cancer care as it was responsible for delaying diagnosis, halting clinical trials, decreasing the capacity for cancer-related surgery, pushing towards the usage of more convenient treatment regimens versus optimal regimens, and leading to a general reprioritization of resources which may have impacted disease follow up. Moreover, cancer screening and treatment delivery has been subjected to significant delays (13). The net effect was a significant increase in cancer-related mortality (14); delays of as little as 4 weeks for surgical, systemic, or radiotherapeutic regimens were associated with increased mortality among patients with cancer. Interestingly, there exists no literature denoting such effects or their magnitude on immigrants, populations residing in areas of conflict, and refugees.

Cancer is the second most common cause of death in Jordan and still represents a significant challenge to the burdened healthcare systems in the Middle East (15, 16). King Hussein Cancer Center (KHCC) is a comprehensive cancer care facility in the region and is the only hospital solely dedicated to cancer management in Jordan, providing treatment for more than 4,000 new patients and 110,000 patients every year (17). On average, KHCC treated patients with cancer from areas of conflict, which represented 25% to 30% of all new cases diagnosed and managed each year (Center’s Cancer Registry). Cancer care for refugees in Jordan remains substandard and heavily fragmented because of the finite financial support and limited access. In addition, there is a lack of information about cancer in areas of conflict populations in low-income and middle-income countries with very little data on cancer surveillance, registries, patterns, and outcomes in populations at risk (18, 19). Epidemiological studies on this topic are also scarce and were not undertaken in many conflict-affected countries before their crises. Cancer inequalities persists within areas of conflict due to the rapid rates of growth of population in these areas, pre-mature war-related deaths, and financial hardships (7).

In light of the above, the study aims to investigate the impact of COVID-19 on cancer care among patients from areas of conflict, seeking treatment at KHCC for 2018-2021.

## 2 Materials and methods

We performed a retrospective chart review of all non-Jordanian cancer patients visiting KHCC between January 2018 to December 2021. The patients were defined as those being treated at KHCC due to cancer care inaccessibility in their home country due to conflict. Areas of conflict (e.g., wars, political unrest) included Iraq, Libya, Palestine, Sudan, and Syria. From KHCC’s Cancer Registry, which was established in 2006, the following was extracted for all potential patients: date at first contact, age at diagnosis, biological sex, cancer site, cancer histopathology, treatment type and duration, and SEER summary stage.

The data was analyzed using SPSS version 23. Categorical data were presented as frequencies [n (%)], while continuous data were reported as means ± standard deviations. Demographic and clinical data were described for the entire cohort and then stratified according to age group (adults vs. pediatrics, cut-off is 18 years of age), biological sex (male vs. female), year of first contact (i.e., 2018, 2019, 2020, 2021), and area of conflict (i.e., Iraq, Libya, Palestine, Sudan, Syria). For each stratum, the following was reported: frequency of cancer sites, SEER stage, treatment modalities, and mean age at diagnosis. Mean differences between certain subgroups in terms of continuous variables were examined using t-test and ANOVA.

The impact of COVID-19 on the number of admitted patients during the timeframe between 2018 and 2021 was modeled using Interrupted Time Series (ITS) analysis. The ITS model incorporated 3 distinct time frames which are a result of (1) the start of the national COVID-19 lockdown and (2) the end of all COVID-19 restrictions. Those time frames include: pre-COVID-19 era (January 2018 – February 2020), COVID-19 lockdown era (March 2020 – August 2021), and post-COVID-19 era (September 2021 – December 2021). Pre-COVID-19 data was collected as early as 2018 as to provide the model with a large enough number of observations for trend assessment. The Autoregressive Integrated Moving Average (ARIMA) model was used to evaluate the impact of COVID-19 lockdown on patients with cancer from areas of conflict. The SPSS Expert Modeler produced a best-fitting ARIMA model of (0,1,0). The fitness of the model and autocorrelations were assessed by the Ljung-Box Q test (p = 0.582). The model stationary R-squared and R-squared values were 0.759 and 0.754, respectively. All statistical tests were conducted with a 95% confidence interval and a 5% error margin. A p-value of less than 0.05 was considered statistically significant.

## 3 Results

### 3.1 Demographic characteristics

We included a total of 3317 patients from areas of conflict with cancer treated at KHCC from 2018 to 2021. A total of 1086 (32.7%), 1119 (33.7%), 489 (14.7%), and 623 (18.8%) patients presented in 2018, 2019, 2020, and 2021, respectively. Among these, 1546 were males (46.6%) while 1771 (53.4%) were females. A total of 2833 patients were adults (i.e., 18 years of age and older) and 484 (14.6%) were children and adolescents (i.e., younger than 18 years of age). In terms of nationality, 812 (24.5%) were Iraqi, 1149 (34.6%) were Libyan, 821 (24.8%) were Palestinian, 29 (0.9%) were Sudanese, and 506 (15.3%) were Syrians.

### 3.2 Time trends

**Figure 1:**
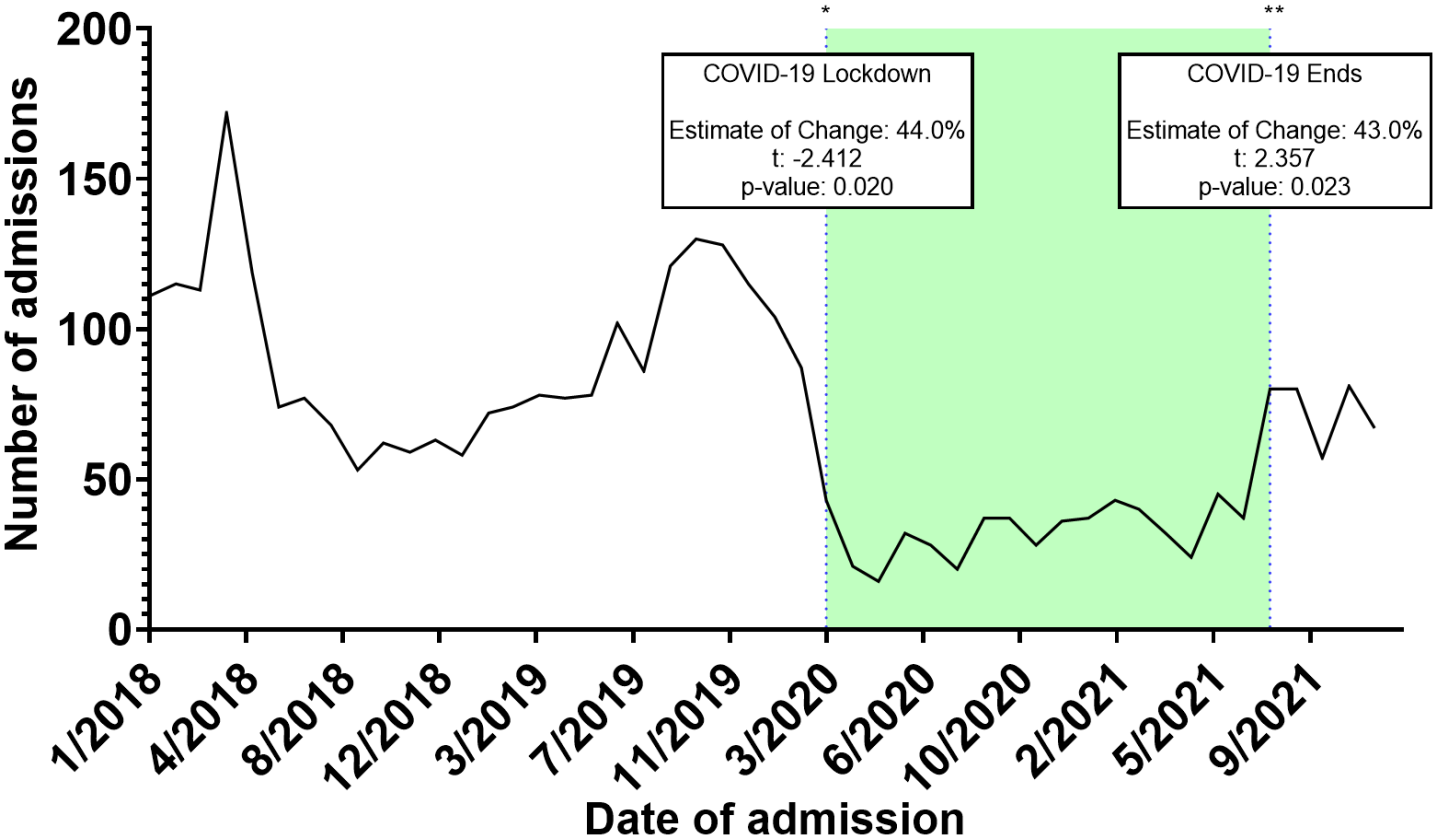
Interrupted time series (ITS) analysis of different COVID-19 related events on the admissions trends of patients from areas of conflict.

**Figure 2:**
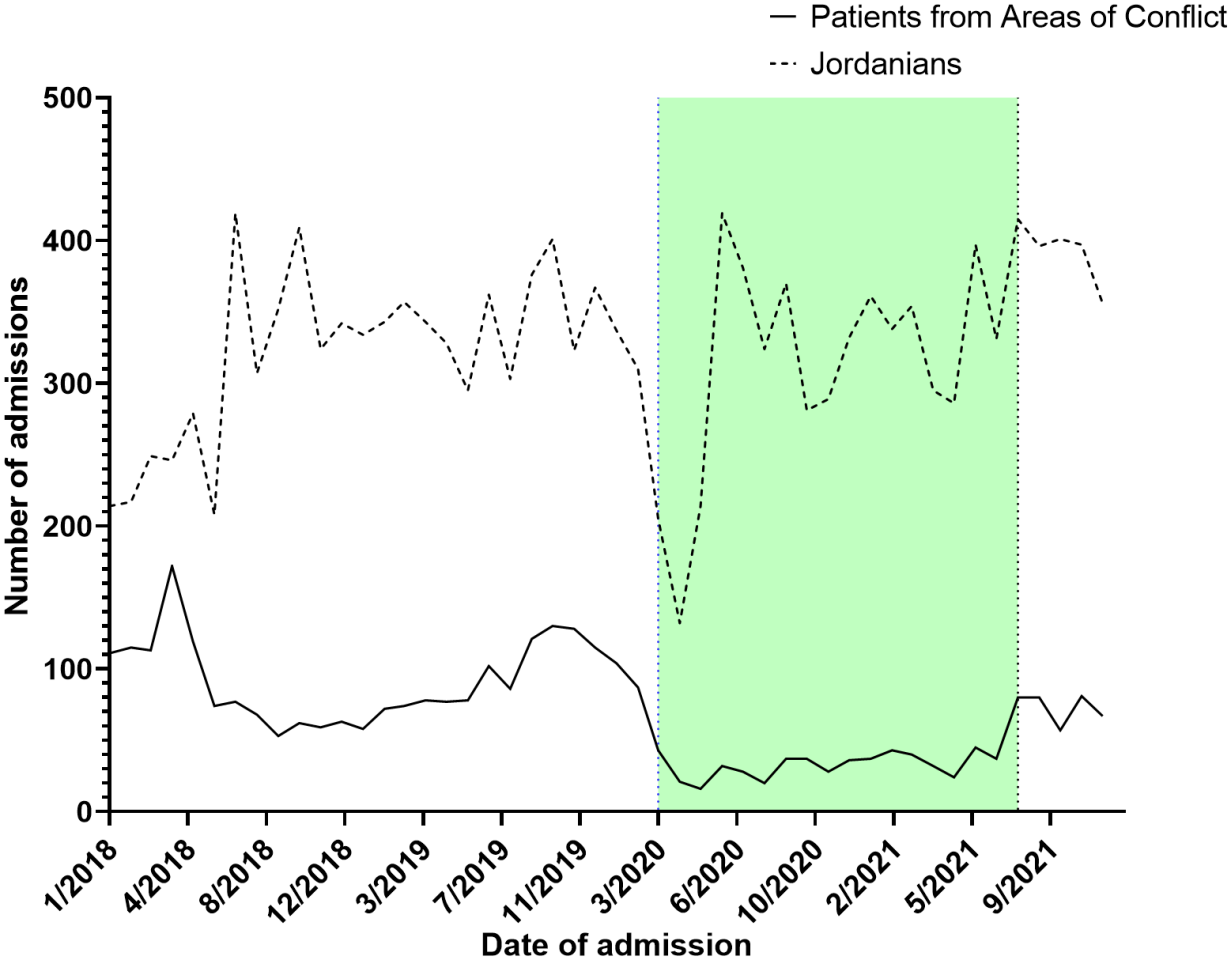
A parallel comparison between admission trends between Jordanian patients with cancer and patients from areas of conflict from 2018 to 2021.

**Figure 3:**
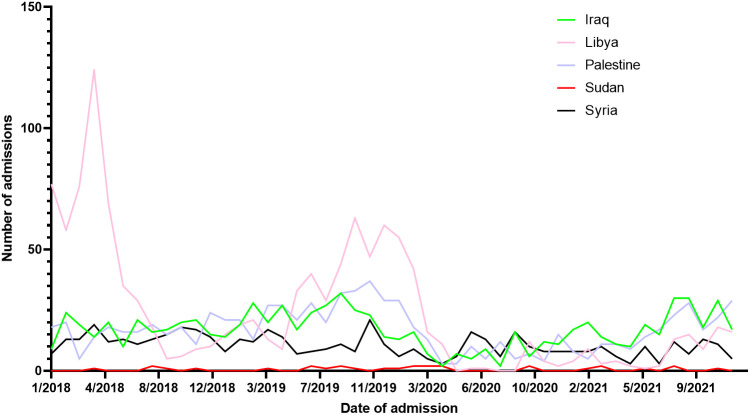
Admissions trends of patients from areas of conflict stratified by nationality from 2018 to 2021.

Throughout 2018, a median of 75.5 [62.2 – 114.5] admissions was recorded ranging from a peak of 174.0 during April to a trough of 53.0 during September. Similarly, across 2019, a median of 82.0 [74.7 – 119.5] admissions were recorded ranging from 130.0 in October to 58 in January of the same year. In 2020 (start of the COVID-19 pandemic), median admissions were reduced to a median of 34.0 [22.7 – 41.5] ranging from 16.0 in May to 104.0 in January (before the actual COVID-19 spread in Jordan). Between February of 2020 and March of 2020 (COVID-19 lockdown started), admissions dropped from 87.0 to 43.0. A trend of sub-50 admissions continued throughout the year, until August of 2021 (End of COVID-19 restrictions) when admissions reached 80.0 signifying an upward trend. ITS analysis demonstrated that the start of the COVID-19 lockdown significantly decreased admissions by 44.0%, while the end of the COVID-19 restriction significantly improved admissions by 43.0% (**Figure 1**). From 2018 to 2021, a total of 15,619 Jordanian patients were admitted to KHCC. With the start of the COVID-19 lockdown, the number of Jordanian patients dropped from 310 in February to 205 in March. These numbers reached their deepest trough in April (n = 132). **Figure 2** demonstrates the time trends between Jordanian and patients from areas of conflict. The number of admissions from 2018 to 2021 stratified by nationality is demonstrated in **Figure 3**.

### 3.3 Clinical characteristics

Across our cohort, the mean age at diagnosis was 45.1 ± 20.6 years. Participants presenting in 2019 had the highest mean age at diagnosis (46.6 ± 19.8), while those presenting in 2020 had the lowest (42.0 ± 22.5). Sudanese and Iraqi patients had the highest mean age at diagnosis with 51.3 ± 16.3 and 49.7 ± 19.5 years, respectively. On the other hand, Syrian refugee patients had the lowest mean age at diagnosis at 37.6 ± 21.7 years. Statistical differences between sexes in terms of age at diagnosis were statistically insignificant (44.9 ± 22.4 (Males) vs. 45.2 ± 19.0 (Females); p = 0.558). Of the reviewed patients, 1597 (48.1%) underwent surgery, 929 (28.0%) received radiation therapy, and 1947 (58.7%) received chemotherapy. A total of 287 (8.7%) experienced tumor recurrence. **Tables 1**, **2** describe the site-specific frequencies of cancer among patients stratified by nationality and year of contact.

**Table 1:** Site-specific frequencies of cancers in patients from areas of conflict who presented to the King Hussein Cancer Center between 2018 and 2021 stratified by nationality.

Site	Total (n = 3317) *n* (%)	Iraq (n = 812) *n* (%)	Libya (n = 1149) *n* (%)	Palestine (n = 821) *n* (%)	Sudan (n = 29) *n* (%)	Syria (n = 506) *n* (%)
Breast	710 (21.4)	227 (28.0)	206 (17.9)	169 (20.6)	5 (17.2)	103 (20.4)
Hemolymphoid	599 (18.1)	86 (10.6)	233 (20.3)	162 (19.7)	5 (17.2)	113 (22.3)
Digestive System	547 (16.5)	143 (17.6)	212 (18.5)	128 (15.6)	5 (17.2)	59 (11.7)
Lung and Pleura	196 (5.9)	50 (6.2)	70 (6.1)	46 (5.6)	4 (13.8)	26 (5.1)
Female Genital System	182 (5.5)	53 (6.5)	67 (5.8)	32 (3.9)	3 (10.3)	27 (5.3)
Endocrine System	180 (5.4)	45 (5.5)	43 (3.7)	62 (7.6)	2 (6.9)	28 (5.5)
Urinary system	168 (5.1)	29 (3.6)	57 (5.0)	49 (6.0)	1 (3.4)	32 (6.3)
Male Genital System	163 (4.9)	40 (4.9)	66 (5.7)	36 (4.4)	1 (3.4)	20 (4.0)
Brain	141 (4.3)	23 (2.8)	61 (5.3)	36 (4.4)	0 (0.0)	21 (4.2)
Bone and Soft Tissue	140 (4.2)	37 (4.6)	44 (3.8)	28 (3.4)	2 (6.9)	29 (5.7)
Head and Neck	108 (3.3)	22 (2.7)	45 (3.9)	28 (3.4)	1 (3.4)	12 (2.4)
Eye	63 (1.9)	21 (2.6)	16 (1.4)	10 (1.2)	0 (0.0)	16 (3.2)
Larynx	50 (1.5)	15 (1.8)	15 (1.3)	11 (1.3)	0 (0.0)	9 (1.8)
Skin	36 (1.1)	11 (1.4)	6 (0.5)	14 (1.7)	0 (0.0)	5 (1.0)
Unknown	17 (0.5)	7 (0.9)	4 (0.3)	5 (0.6)	0 (0.0)	1 (0.2)
Nose and Ear	17 (0.5)	3 (0.4)	4 (0.3)	5 (0.6)	0 (0.0)	5 (1.0)

**Table 2:** Site-specific frequencies of cancers in patients from areas of conflict who presented to the King Hussein Cancer Center between 2018 and 2021 stratified by year of first contact.

Site	Total (n = 3317) *n* (%)	2018 (n = 1086) *n* (%)	2019 (n = 1119) *n* (%)	2020 (n = 489) *n* (%)	2021 (n = 623) *n* (%)
Breast	710 (21.4)	224 (20.6)	257 (23.0)	111 (22.7)	118 (18.9)
Hemolymphoid	599 (18.1)	196 (18.0)	198 (17.7)	90 (18.4)	115 (18.5)
Digestive System	547 (16.5)	198 (18.2)	177 (15.8)	69 (14.1)	103 (16.5)
Lung and Pleura	196 (5.9)	54 (5.0)	75 (6.7)	28 (5.7)	39 (6.3)
Female Genital System	182 (5.5)	72 (6.6)	57 (5.1)	24 (4.9)	29 (4.7)
Endocrine System	180 (5.4)	56 (5.2)	52 (4.6)	30 (6.1)	42 (6.7)
Urinary system	168 (5.1)	53 (4.9)	50 (4.5)	25 (5.1)	40 (6.4)
Male Genital System	163 (4.9)	55 (5.1)	59 (5.3)	23 (4.7)	26 (4.2)
Brain	141 (4.3)	46 (4.2)	45 (4.0)	26 (5.3)	24 (3.9)
Bone and Soft Tissue	140 (4.2)	44 (4.1)	44 (3.9)	24 (4.9)	28 (4.5)
Head and Neck	108 (3.3)	34 (3.1)	40 (3.6)	16 (3.3)	18 (2.9)
Eye	63 (1.9)	15 (1.4)	22 (2.0)	8 (1.6)	18 (2.9)
Larynx	50 (1.5)	14 (1.3)	15 (1.3)	7 (1.4)	14 (2.20)
Skin	36 (1.1)	15 (1.4)	14 (1.3)	5 (1.0)	2 (0.3)
Unknown	17 (0.5)	4 (0.4)	8 (0.7)	2 (0.4)	3 (0.5)
Nose and Ear	17 (0.5)	6 (0.6)	6 (0.5)	1 (0.2)	4 (0.6)

**Figure 4:**
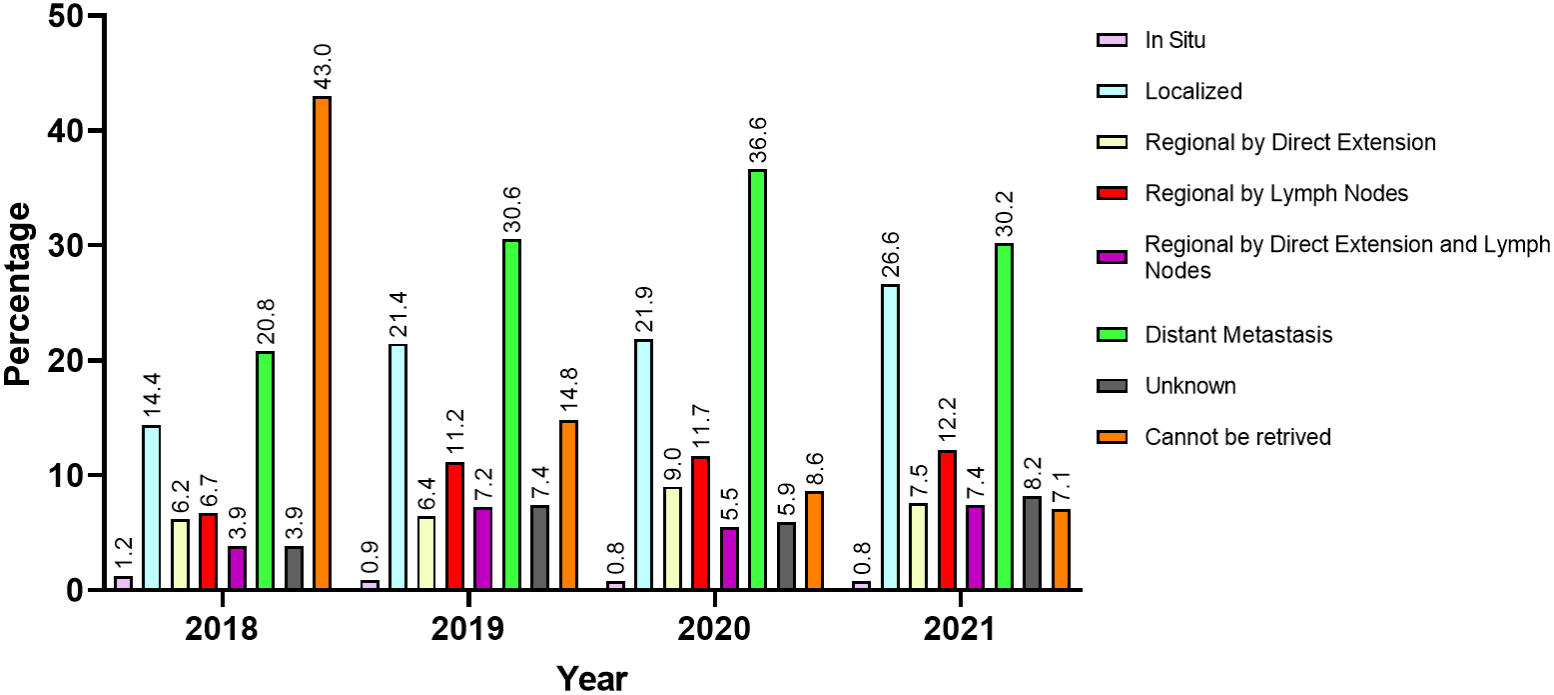
The SEER summary stage at presentation of patients from areas of conflict who presented to the King Hussein Cancer Center between 2018 and 2021 stratified by year of first contact.

For a total of 924 patients (27.9%), the SEER summary stage was unknown or could not be retrieved. Among those with available SEER stages, 935 (28.2%) had distant metastasis, 669 (20.2%) had localized disease, and 757 (22.8%) had regionally extending disease by direct extension or lymph nodes metastasis or both. **Table 3** and **Figure 4** describe the available SEER summary stage frequencies of all cases among patients stratified by nationality and year of contact. Clinical characteristics for both site-specific cancer frequencies and SEER summary stages stratified by biological sex and age can be found within **Supplementary Tables 1, 2**.

**Table 3:** The SEER summary stage at presentation of patients from areas of conflict who presented to the King Hussein Cancer Center between 2018 and 2021 stratified by nationality.

Site	Total (n = 3317) *n* (%)	Iraq (n = 812) *n* (%)	Libya (n = 1149) *n* (%)	Palestine (n = 821) *n* (%)	Sudan (n = 29) *n* (%)	Syria (n = 506) *n* (%)
*In situ*	32 (1.0)	5 (0.6)	6 (0.5)	15 (1.8)	0 (0.0)	6 (1.2)
Localized	669 (20.2)	219 (27.0)	167 (14.5)	178 (21.7)	4 (13.8)	101 (20.0)
Regional by direct extension	230 (6.9)	54 (6.7)	77 (6.7)	49 (6.0)	2 (6.9)	48 (9.5)
Regional to lymph nodes	331 (10.0)	102 (12.6)	68 (5.9)	99 (12.1)	2 (6.9)	60 (11.9)
Regional by direct extension and lymph nodes	196 (5.9)	49 (6.0)	60 (5.2)	54 (6.6)	5 (17.2)	28 (5.5)
Distant	935 (28.2)	195 (24.0)	320 (27.9)	237 (28.9)	13 (44.8)	170 (33.6)
Unknown	205 (6.2)	68 (8.4)	37 (3.2)	66 (8.0)	2 (6.9)	32 (6.3)
None	719 (21.7)	120 (14.8)	414 (36.0)	123 (15.0)	1 (3.4)	61 (12.1)

## 4 Discussion

In this observational study, we reviewed clinical records of 3317 patients from countries with conflict who received cancer care at KHCC during the years 2018 – 2021. Our study is the first to report changes on the trends of these cancer cases in light of the COVID-19 pandemic. The data herein showcases that breast cancer was the most common in the female cohort across all nationalities while hemolymphoid malignancies were most common among their male counterparts except for Iraqi and Sudanese male patients. Cancers of the digestive system ranked first among male Iraqi and Sudanese patients and second among Palestinian, Syrian, and Libyan male, and Libyan female patients. Hematolymphoid cancers were the second most common among Palestinian and Syrian female patients. Moreover, we have demonstrated that a significant number of these patients presented with distant metastasis irrespective of gender, age, or nationality. Overall, the frequency of presentation with advanced stage cancer (i.e., distant metastasis) increased amidst the COVID-19 lockdown.

We have observed a significant decline of 44% of cancer cases after the onset of the COVID-19 lockdown around March 2020. These effects were counterbalanced by the lifting of COVID-19 social and travel restrictions on August 2021 as it was associated with a 43.0% increase in cancer cases. Our results are echoed within the literature as an investigation of cancer patients hospitalized at the Massachusetts General Hospital demonstrated a reduction of admissions reaching as high as 45.4%, particularly during April 2020 (20). The study also shows that the inpatient census increased following the ease of the COVID-19 surge within the Boston region. Overall, the effect of COVID-19 on cancer diagnosis, screening, and treatment is well documented throughout literature (21–25). Our results further add to the growing body of literature by demonstrating the overall impact of these changes within the context of cancer patients from areas of conflict.

Interestingly, when comparing our target population to Jordanian patients with cancer treated at KHCC, both populations experienced a significant decrease in admissions around March 2020. However, for patients from areas of conflict, it took around 18 months for their numbers to recover to pre-COVID-19 rates. This was in contrast with Jordanians, whose numbers stabilized back to their pre-COVID-19 thresholds in a matter of few months. This observation is expected as Jordanian patients with cancer did not experience the travel restrictions and delays associated with COVID-19 control policies.

The COVID-19 pandemic has been a particularly challenging time for patients with cancer. On one hand, those patients are often vulnerable because of their immunocompromised state, while increasing evidence showed that patients with cancer are at a higher risk of COVID-19 infection (26). This dilemma may have predisposed patients to be more reluctant to consult their primary physician with regard to their disease or scheduled follow-ups (27–29). As for cancer care delivery, emergency planning strategies and policies during the onset of the pandemic were focused on relocating all healthcare resources to the containment of the virus, including those dedicated to cancer services (30). Data from 356 centers across 54 countries showed the devastating impact of COVID-19 on cancer healthcare as it reported interruption of cancer-specific care ranging from 36.5% to 80.0%. These manifested as treatment delays, missing treatment, and entire obstruction of cancer care delivery due to lack of capacity (31).

Not only did the COVID-19 pandemic affect the activity of general and specialized practice, but it also disrupted the coordination between different practices and hospitals which led to decreased referrals for possible cases of cancer to specialized centers (32, 33). This is especially important when considering that KHCC is Jordan’s only specialized tertiary center where the cornerstone multi-modal interventions are available (17). Studying the impact of COVID-19 on cancer care outlines the inflection at which new cases are presented to healthcare systems after the alleviation of restrictions. This also poses questions about whether the pandemic has affected diagnostic patterns and stages at diagnosis, therefore, outcomes. A national, population-based study in the United Kingdom demonstrated that the delays in diagnosis of four tumors associated with the COVID-19 lockdown (breast, colorectal, lung, and esophageal), led to over 3,000 avoidable deaths and more than 59,000 years of life lost (34). It should be noted that the impact of these delays on patient outcomes is not entirely known. These challenges are especially important in the context of underserved cancer patients in areas of conflict seeking treatment at our center, further preventing them from accessing appropriate cancer care. The noted interruptions in cancer care delivery across the healthcare systems could have deleterious effects on patients’ outcomes on aspects such as increased tumor size, higher chances of node invasion at diagnosis, along with increased rates of mortality on both short and long term periods (35–37). These patterns, which align with our results, come as no surprise, as policies during the early months of the pandemic were primarily executed to address the complications of the early aggressive COVID-19 strain on the vulnerable public while ensuring the safety of healthcare providers.

Being a leading cause of mortality, cancer exerts a colossal burden on global healthcare. Also, it is associated with an ever-rising interest from international communities to provide more effective and efficient methods of treatment, diagnosis, screening, and prevention; however, cancer care is still widely inequitable around the world. This is especially evident when observing underprivileged populations such as those residing in politically unstable regions (38). Such populations often encounter many hardships that delay diagnosis, impede care delivery, and render screening almost impossible. The detrimental effects of such challenges are reflected upon patients as they often present with advanced stages which generally lead to poorer survival and quality of life outcomes (39–41). Our results delineate that more than a quarter of patients had already presented with distant metastasis during the pandemic; a trend which only increased in frequency after the implementation of the COVID-19 lockdown in 2020, as more than a third of patients presented with distant metastasis.

Studies exclusively studying the Syrian populations in Turkey reported that the majority of Syrian refugees present with breast cancer and advanced stages. On the other hand, Jordanian reports showed a dominance of localized breast cancer cases among Syrian refugees (10, 42, 43). The UNHCR demonstrated that, in general, refugees in formal camps have very poor survival outcomes even when diagnosed early with cancer. This is mainly due to the inadequacy of their received treatment plans as well as the overall poor health infrastructure of their camps which are primarily tailored to treating communicable diseases. Furthermore, the clinical and surgical services in those camps are geared towards ‘classical’ refugee health, mainly dealing with trauma and benign diseases (18, 44).

Here, we note the importance of National Cancer Control Plans (NCCPs) in designing policies that aim to reduce the burden of cancer in Jordan among Jordanians in general and among refugee populations in specific. In addition to investing in cost-effective cancer screening measures, evidence shows that primary cancer prevention through controlling cancer risk factors, such as smoking, diet (e.g., obesity), and environmental pollution are critical in reducing cancer incidence and cancer-related healthcare costs (45, 46). According to expert stakeholders, designing holistic cancer control plans, which include the entire cancer journey spectrum from early detection to survival and palliation, is ultimately cost-effective in the long run as it decreases treatment costs for advanced and highly sophisticated cases (47). The World Health Organization laid out in its 2019 cancer report that for every 1 USD invested in those programs, there is a 2.30 USD of direct productivity return and 9.50 USD of social productivity return (48).

Estimating the true burden of cancer in refugees is imperative to the design of cost-effective cancer control strategies. However, such a process is hindered by a variety of factors. Firstly, cancer is not one disease entity but rather a spectrum of related diseases, each with a unique set of risk factors and clinical behaviors. Secondly, the availability of data poses an important challenge as patients coming from areas of conflict including refugees often come from lower income settings where registries are inadequate or absent (49). These limitations are augmented by many folds when considering the significant rates of attrition of these patients due to poor economic standards. This loss of data makes a reliable calculation of survival rates extremely burdensome, let alone controlling it for possible confounders. Thirdly, factors affecting cancer care among this particular group of patients are dynamic and multileveled ranging from differences in risk exposure, and access to healthcare, to behavioral/psychosocial changes (50). The literature highlights the paucity of epidemiological data on cancer patients in low-to-middle-income countries and their associated changes in risk factors, presentation delays, and access to care (50). In addition, the aforementioned literature is often methodologically weak and at times contradictory.

Our paper falls prey to a number of limitations. Firstly, the study’s retrospective design may have limited the range of extracted variables. Secondly, due to the participants’ temporary residence within Jordan, maintenance of follow-up is often hindered and may have restricted our ability to reliably calculate and compare outcome measures (e.g., overall survival). Finally, staging data per the 2018 SEER guidelines were not available for all patients.

## 5 Conclusion

We have demonstrated that the restrictions associated with COVID-19 had significantly affected the number of admissions of patients from areas of conflict within the MENA region. This effect, manifesting as missed treatments or treatment delays, may be projected as worse survival outcomes for a population that is already vulnerable, both clinically, and socially. Therefore, concerned bodies are encouraged to form policies that aim to sustain the well-being of those patients during times of extreme hazard.

## Data availability statement

All data/data sets associated with this project can be requested from the corresponding author at a reasonable request.

## Ethics statement

The studies involving human participants were reviewed and approved by the Institutional Review Board of the KHCC (Amman, Jordan), protocol number 22KHCC171. Written informed consent from the participants’ legal guardian/next of kin was not required to participate in this study in accordance with the national legislation and the institutional requirements.

## Author contributions

Conceptualization: MA-H and AM. Methodology: MA-H and AA-A. Formal analysis: AA-A and LA-H. Writing – Original Draft: All authors. Writing – Review & Editing: All authors. Supervision: MA-H and AM. All authors contributed to the article and approved the submitted version.
