# Supplementary material for: Investigating the impact of COVID-19 on patients with cancer from areas of conflict within the MENA region treated at King Hussein Cancer Center
Source: Front Oncol. 2023 Feb 23;13:1088000. doi: 10.3389/fonc.2023.1088000 (PMC9995942; doi:10.3389/fonc.2023.1088000)
Supplement: Supplementary file 1 [file Table_1.pdf]

Supplementary Table 1

Site	**Total** (n = 3317) *n* (%)	Female (n = 1771) *n* (%)	Male (n = 1546) *n* (%)	**Adults** (n = 2833) *n* (%)	Adult females (n = 1566) *n* (%)	Adult males (n = 1267) *n* (%)	**Children and adolescents** (n = 484) *n* (%)	Girls and female adolescents (n = 205) *n* (%)	Boys and male adolescents (n = 279) *n* (%)
Breast	710 (21.4)	700 (39.5)	10 (0.6)	710 (25.1)	700 (44.7)	10 (0.8)	0 (0.0)	0 (0.0)	0 (0.0)
Hemolymphoid	599 (18.1)	236 (13.3)	363 (23.5)	388 (13.7)	154 (9.8)	234 (18.5)	211 (43.6)	82 (40.0)	129 (46.2)
Digestive System	547 (16.5)	245 (13.8)	302 (19.5)	531 (18.7)	238 (15.2)	293 (23.1)	16 (3.3)	7 (3.4)	9 (3.2)
Lung and Pleura	196 (5.9)	39 (2.2)	157 (10.2)	193 (6.8)	39 (2.5)	154 (12.2)	3 (0.6)	0 (0.0)	3 (1.1)
Female Genital System	182 (5.5)	182 (10.3)	0 (0.0)	173 (6.1)	173 (11.0)	0 (0.0)	9 (1.9)	9 (4.4)	0 (0.0)
Endocrine System	180 (5.4)	113 (6.4)	67 (4.3)	148 (5.2)	99 (6.3)	49 (3.9)	32 (6.6)	14 (6.8)	18 (6.5)
Urinary system	168 (5.1)	47 (2.7)	121 (7.8)	139 (4.9)	29 (1.9)	110 (8.7)	29 (6.0)	18 (8.8)	11 (3.9)
Male Genital System	163 (4.9)	0 (0.0)	163 (10.5)	156 (5.5)	0 (0.0)	156 (12.3)	7 (1.4)	0 (0.0)	7 (2.5)
Brain	141 (4.3)	48 (2.7)	93 (6.0)	79 (2.8)	23 (1.5)	56 (4.4)	62 (12.8)	25 (12.2)	37 (13.3)
Bone and Soft Tissue	140 (4.2)	63 (3.6)	77 (5.0)	77 (2.7)	34 (2.2)	43 (3.4)	63 (13.0)	29 (14.1)	34 (12.2)
Head and Neck	108 (3.3)	39 (2.2)	69 (4.5)	101 (3.6)	37 (2.4)	64 (5.1)	7 (1.4)	2 (1.0)	5 (1.8)
Eye	63 (1.9)	30 (1.7)	33 (2.1)	23 (0.8)	13 (0.8)	10 (0.8)	40 (8.3)	17 (8.3)	23 (8.2)
Larynx	50 (1.5)	6 (0.3)	44 (2.8)	49 (1.7)	5 (0.3)	44 (3.5)	1 (0.2)	1 (0.5)	0 (0.0)
Skin	36 (1.1)	13 (0.7)	23 (1.5)	34 (1.2)	12 (0.8)	22 (1.7)	2 (0.4)	1 (0.5)	1 (0.4)
Unknown	17 (0.5)	6 (0.3)	11 (0.7)	17 (0.6)	6 (0.4)	11 (0.9)	0 (0.0)	0 (0.0)	(0.0)
Nose and Ear	17 (0.5)	4 (0.2)	13 (0.8)	15 (0.5)	4 (0.3)	11 (0.9)	2 (0.4)	0 (0.0)	2 (0.7)
