# Supplementary material for: Investigating the impact of COVID-19 on patients with cancer from areas of conflict within the MENA region treated at King Hussein Cancer Center
Source: Front Oncol. 2023 Feb 23;13:1088000. doi: 10.3389/fonc.2023.1088000 (PMC9995942; doi:10.3389/fonc.2023.1088000)
Supplement: Supplementary file 2 [file Table_2.pdf]

Supplementary Table 2

Stage	**Total** (n = 3317) *n* (%)	Female (n = 1771) *n* (%)	Males (n = 1546) *n* (%)	Adult females (n = 1267) *n* (%)	Adult males (n = 1566) *n* (%)	Girls and female adolescents (n = 279) *n* (%)	Boys and male adolescents (n = 205) *n* (%)
In situ	32 (1.0)	17 (1.0)	15 (1.0)	15 (1.2)	17 (1.1)	0 (0.0)	0 (0.0)
Localized	669 (20.2)	375 (21.2)	294 (19.0)	220 (17.4)	321 (20.5)	74 (26.5)	54 (26.3)
Regional by direct extension	230 (6.9)	98 (5.5)	132 (8.5)	103 (8.1)	77 (4.9)	29 (10.4)	21 (10.2)
Regional to lymph nodes	331 (10.0)	269 (15.2)	62 (4.0)	59 (4.7)	266 (17.0)	3 (1.1)	3 (1.5)
Regional both by direct extension and lymph nodes	196 (5.9)	89 (5.0)	107 (6.9)	103 (8.1)	85 (5.4)	4 (1.4)	4 (2.0)
Distant	935 (28.2)	415 (23.4)	520 (33.6)	410 (32.4)	339 (21.6)	110 (39.4)	76 (37.1)
Unknown	205 (6.2)	106 (6.0)	99 (6.4)	86 (6.8)	97 (6.2)	13 (4.7)	9 (4.4)
None	719 (21.7)	402 (22.7)	317 (20.5)	271 (21.4)	364 (23.2)	46 (16.5)	38 (18.5)
